# Targeting Ferroptosis for Lung Diseases: Exploring Novel Strategies in Ferroptosis-Associated Mechanisms

**DOI:** 10.1155/2021/1098970

**Published:** 2021-10-06

**Authors:** Tian-Liang Ma, Yong Zhou, Ci Wang, Lu Wang, Jing-Xian Chen, Hui-Hui Yang, Chen-Yu Zhang, Yong Zhou, Cha-Xiang Guan

**Affiliations:** ^1^Department of Physiology, School of Basic Medical Science, Central South University, Changsha, Hunan 410078, China; ^2^Department of Orthopedics, Xiangya Hospital, Central South University, Changsha, Hunan 410008, China; ^3^Hunan Engineering Research Center of Biomedical Metal and Ceramic Implants, Xiangya Hospital, Central South University, Changsha, Hunan 410008, China; ^4^Department of Cardiology, The Second Xiangya Hospital, Central South University, Changsha, Hunan 410011, China; ^5^Department of Cardiovascular Medicine, First Teaching Hospital of Tianjin University of Traditional Chinese Medicine, National Clinical Research Center for Chinese Medicine Acupuncture and Moxibustion, Tianjin University of Traditional Chinese Medicine, Tianjin 300381, China

## Abstract

Ferroptosis is an iron-dependent regulated necrosis characterized by the peroxidation damage of lipid molecular containing unsaturated fatty acid long chain on the cell membrane or organelle membrane after cellular deactivation restitution system, resulting in the cell membrane rupture. Ferroptosis is biochemically and morphologically distinct and disparate from other forms of regulated cell death. Recently, mounting studies have investigated the mechanism of ferroptosis, and numerous proteins play vital roles in regulating ferroptosis. With detailed studies, emerging evidence indicates that ferroptosis is found in multiple lung diseases, demonstrating that ferroptosis appears to be particularly important for lung diseases. The mounting interest in ferroptosis drugs specifically targeting the ferroptosis mechanism holds substantial therapeutic promise in lung diseases. The present review emphatically summarizes the functions and integrated molecular mechanisms of ferroptosis in various lung diseases, proposing that multiangle regulation of ferroptosis might be a promising strategy for the clinical treatment of lung diseases.

## 1. Background

The systematic analysis for the Global Burden of Disease Study 2017 indicates that lung cancer and chronic obstructive pulmonary disease (COPD) are the leading causes of death worldwide [1]. With the outbreak of the 2019 coronavirus disease (COVID-19) in 2020, the surge of confirmed cases poses a severe threat to public health globally. The pathogenesis and epidemiological characteristics of severe acute respiratory syndrome coronavirus-2 (SARS-CoV-2) have recently attracted considerable attention [2]. Ferroptosis is a new form of iron-dependent regulated cell death (RCD), different from necrosis, apoptosis, autophagy, necroptosis, and pyroptosis [3]. As a stress response, ferroptosis mediates the occurrence and development of neurological diseases, tumors, renal injury, ischemia-reperfusion (I/R) injury, and lung diseases [4–6]. Recent studies have suggested that various lung diseases display ferroptosis of lung cells and progressive damage of lung function in the process of chronic development, which is life-threatening ultimately. The role and mechanism of ferroptosis in lung diseases have attracted much attention recently [7]. In the studies of COVID-19, acute lung injury (ALI), pulmonary fibrosis (PF), COPD, lung cancer, and other lung diseases; unstable iron accumulation; and enhanced lipid peroxidation (LPO) are observed in lung cells, accompanied by the downregulation of glutathione peroxidase 4 (GPX4), suggesting that ferroptosis in lung tissue plays a vital role in various lung diseases [8–11]. Thus, it is imperative to investigate further the regulatory mechanism of ferroptosis and its role in lung diseases. Targeting ferroptosis in lung cells has broad prospects for lung diseases therapy.

## 2. Introduction

Ferroptosis is iron-catalyzed regulated necrosis, and it occurs when the intracellular antioxidant systems are inhibited by pharmacological inhibition or genetic knockdown, such as GPX4, ferroptosis suppressor protein 1 (FSP1), cystine/glutamate antiporter SLC7A11 (also commonly known as xCT), and guanosine triphosphate cyclohydrolase 1 (GCH1). The nature of ferroptosis is the peroxidation damage of the cell membrane or organelle membrane caused by the inactivation of the intracellular reduction system, resulting in cell membrane rupture [12, 13]. Ferroptosis requires regulation of other intracellular signaling molecules, as exemplified by protein 53 (p53), nuclear factor erythroid-2-related factor 2 (Nrf2), and mitogen-activated protein kinase (MAPK) [4, 14, 15]. But the specific mechanism is unclear. Mounting evidence indicates multiple oxidation and antioxidant systems can be activated simultaneously and run in parallel to adjust the ferroptosis threshold. The role of ferroptosis in growth and development has remained largely uncharacterized. Still, the disorder of ferroptosis regulation triggers diseases as stress response and participates in various lung diseases [8–11].

## 3. Mechanism and Indicators of Ferroptosis

### 3.1. Quantitative Variation-Accumulation Period of Iron-Dependent Lipid Peroxides

#### 3.1.1. Abnormal Iron Metabolism

In physiological situations, duodenal cytochrome B (DCYTB), a small intestinal cell membrane reductase, reduces Fe^3+^ to Fe^2+^ and transports Fe^2+^ into intestinal epithelial cells through the divalent metal transporter (DMT1) [16]. Fe^2+^ in intestinal epithelial cells is transported to the blood through membrane iron transporters and is oxidized into Fe^3+^ by ferrous oxidase. In the blood circulation, Fe^3+^ binds to transferrin (TF) and undergoes various tissues and organs [16]. The iron reductase six-transmembrane epithelial antigen of prostate 3 (STEAP3) reduces Fe^3+^ to Fe^2+^ in the endosome. Fe^2+^ is transferred to the labile iron pool through DMT1, and excessive iron is transported to the bloodstream or stored in ferritin [11]. Under pathological conditions, excessive free Fe^2+^ will gather in the cytoplasm (iron overload), and Fe^2+^ produces vast amounts of hydroxyl radicals and reactive oxygen species (ROS) through the Fenton reaction, which destroys the cell membrane, DNA, and proteins in the cells and triggers ferroptosis [17]. The labile iron pool is strictly controlled at multiple levels by the p62/keap1/Nrf2 axis. The upregulation of Nrf2 expression promotes the expression of antioxidant-related genes NAD(P)H:quinone oxidoreductase 1 (NQO1), heme oxygenase 1 (HO-1), and ferritin heavy chain 1 (FTH1), which diminishes the production of free Fe^2+^ and LPO and inhibits the occurrence of ferroptosis [15].

#### 3.1.2. Abnormal Lipid Metabolism

Polyunsaturated fatty acids (PUFAs) are one kind of linear fatty acids with two or more double bonds and 18~22 carbon atoms in length [18], which participate in synthesizing lipid signaling molecules. Moreover, PUFAs are esterified into membrane phospholipids and integrated into the cell membrane to regulate cell membrane fluidity. Most investigators concur that PUFAs are easily oxidized to lipid peroxides to induce ferroptosis in cells because of their unstable double bonds [19]. Acyl-CoA synthetase long-chain family member 4 (ACSL4) and lysophosphatidylcholine acyltransferase 3 (LPCAT3) related to lipid remodeling play a central role in the synthesis of PUFAs. With the knockdown of ACSL4 or LPCAT3, the synthesis of PUFAs decreases, and ferroptosis is significantly inhibited [20]. In addition, lipoxygenases (LOXs) catalyze PUFAs to form lipid hydroperoxides, aggravate toxic LPO, and induce ferroptosis [18].

#### 3.1.3. Mitochondrial Dysfunction

Mitochondria are the prominent organelle of intracellular ROS generation and contribute to the regulation of ferroptosis [21]. Inhibiting the activity of GPX4 by RAS selective lethal 3 (RSL3) results in decreased mitochondrial volume, diminished cristae number, and disruption of the outer membrane [22]. The ferroptosis inducer erastin binds to the voltage-dependent anion channel (VDAC2/3) of the mitochondrial outer membrane, downregulates the mitochondrial outer membrane permeability, reduces the NADH oxidation rate, and promotes ferroptosis [23]. In addition, the inhibition of the mitochondrial tricarboxylic acid (TCA) cycle or electron transport chain (ETC) attenuates mitochondrial membrane potential hyperpolarization and lipid peroxide accumulation [21]. Lowering cysteine (Cys) levels promotes intracellular glutamine catabolism, leading to the expansion of lipid peroxides and ferroptosis [21]. Mitochondrial ferritin (MtFt) stores intracellular free Fe^2+^ and inhibits intracellular iron overload, thereby diminishing LPO [24].

#### 3.1.4. GPX4 and System Xc^−^

With the reductant glutathione (GSH), GPX4 catalyzes lipid hydroperoxide and free hydrogen peroxide to produce lipid alcohols (L-OH) and water, inhibits the accumulation of lipid peroxide, and negatively regulates ferroptosis [25]. GSH engages in the precise regulation of GPX4 activity, which functions as a positive regulator in maintaining intracellular redox balance [26]. Cys is essential to the synthesis of intracellular GSH, and its intracellular content depends on the functional activity of the cystine/glutamate antiporter (system Xc^−^) [27, 28]. When the intracellular Cys content is insufficient, GSH synthesis encounters a barrier and GPX4 activity decreases, resulting in an accumulation of intracellular lipid peroxide [27]. Moreover, the transsulfuration pathway is another important source of intracellular GSH synthesis. Inhibition of transsulfuration is a leading cause of the imbalance of the intracellular antioxidant system [29]. When system Xc^−^ is inhibited by erastin, DJ-1 (also known as Parkinson disease type 7, PARK7) inhibits the interaction between adenosylhomocysteinase like 1 (AHCYL1) and S-adenosyl-L-homocysteine hydrolase (SAHH), maintains the activity of SAHH, and sustains the essential synthesis of Cys [30]. Thus, inhibition of the system Xc^−^ appears to be particularly important for reducing GSH, precisely regulating cells' antioxidant function.

#### 3.1.5. FSP1

FSP1 is a novel coenzyme Q10 (CoQ10) plasma membrane oxidoreductase, protecting cells from glutathione-dependent ferroptosis [31, 32]. FSP1 is localized to the cytoplasmic membrane after acylation modification. It promotes the transformation of CoQ10 to Ubiquinol-10 (CoQH2) with the assistance of NAD(P)H to generate radical-trapping antioxidants (RTA), which prevented the proliferation of LPO [33, 34]. In some cases, FSP1 directly activates the endosomal sorting complex required for transport-III (ESCRT-III) to promote cell membrane repair and inhibit ferroptosis [35]. With detailed studies, increased FSP1 expression reduces LPO and inhibits ferroptosis when GPX4 is inactivated [36]. Consequently, the FSP1/CoQ10/NAD(P)H pathway is an antioxidant system parallel to GPX4, which cooperates with GPX4 and glutathione to upregulate antiferroptotic defense.

#### 3.1.6. GCH1

GCH1 is a rate-limiting enzyme in the synthesis of tetrahydrobiopterin (BH4), which maintains vital antioxidant activity [37, 38]. GCH1 enhances the synthesis of BH4/BH2 to induce lipid remodeling and inhibits phospholipid consumption containing two polyunsaturated fatty acyl groups to diminish ferroptosis [39]. In addition, the overexpression of GCH1 favors the formation of CoQ10 and synergistically exerts the inhibitory effect of ferroptosis. BH4 is a cofactor for dopamine key enzymes that attenuates erastin-induced ferroptosis [40]. The GCH1-BH4-phospholipid axis functions as a negative regulator of ferroptosis by promoting the synthesis of endogenous BH4 and CoQ10 and reducing the formation of lipid peroxides [39].

#### 3.1.7. NADPH

NADPH is a ferroptosis inhibitor produced by the pentose phosphate pathway, which suppresses ferroptosis induced by erastin. The increase of the NADP/NADPH ratio promotes ferroptosis sensitivity, ferroptosis-resistant cell lines are associated with the high expression of NADPH, and NADPH oxidase- (NOX-) mediated NADPH oxidation triggers ferroptosis [41]. Nrf2 is an essential nuclear transcription factor of intracellular antioxidants, enhancing NADPH synthesis by regulating the pentose phosphate pathway [42]. NADPH functions as a positive regulator of the antioxidant activity of GPX4 and FSP1 by regulating electron transport and plays a vital role in attenuating ferroptosis.

#### 3.1.8. Others

Multiple intracellular signaling molecules regulate ferroptosis. For example, p53 is the negative regulator of the system Xc^−^ activity and intracellular antioxidant GSH production. In addition, p53 promotes the expression of spermidine/spermine N1-acetyltransferase (SAT1), increases the expression of arachidonic acid 15-LOX, and accelerates intracellular lipid peroxide accumulation [43]. Heat shock protein beta-1 (HSPB1) is phosphorylated by protein kinase C in Hela cells, blocking cytoskeleton-mediated iron uptake and subsequent ROS production and inhibiting ferroptosis [44]. Nuclear receptor coactivator 4 (NCOA4) is a selective freighter receptor that promotes selective autophagy of ferritin. Knockdown of NCOA4 inhibits ferritin degradation and prevents ferroptosis [45]. High mobility group protein B1 (HMGB1) is another crucial ferroptosis regulator, promoting erastin-induced ROS production through an iron-mediated lysosome pathway. Moreover, HMGB1 increases the level of TFR through the RAS-JNK/p38 pathway and exacerbates intracellular iron overload and lethal accumulation of lipid peroxides [46, 47]. Thus, multiple intracellular signaling molecules participate in ferroptosis, and the specific role and mechanism need to be further elucidated [47].

### 3.2. Quality Change-Oxidative Damage of Liposome Membrane

**Figure 1:**
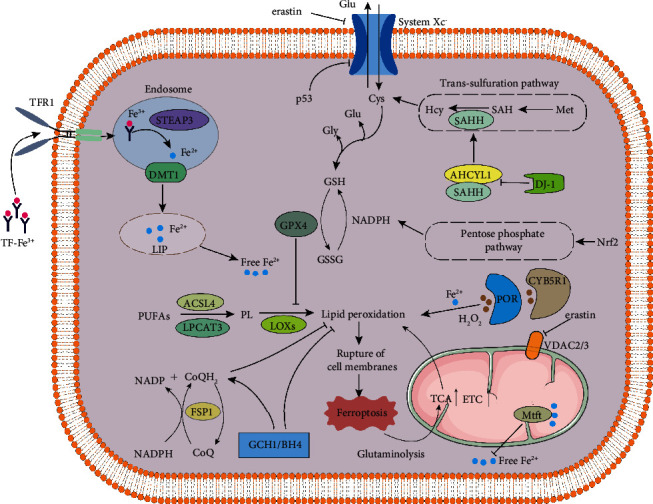


NADPH-cytochrome P450 reductase (POR) and cytochrome b5 reductase (CYB5R1) transmit electrons from NADPH/NADH to oxygen; the generated H_2_O_2_ undergoes Fenton reaction with iron ions to produce hydroxyl radicals, which deprives the hydrogen atoms between the double bonds in the unsaturated fatty acid chain, triggers LPO, and causes membrane damage [48, 49]. Oxidative stress in the process of ferroptosis comes from the “accidental” electron leakage of intracellular oxidoreductase during electron transfer. The intracellular antioxidant systems alleviate oxidative damage under physiological conditions. The cell membrane damage during ferroptosis is a nondependent lipid peroxidation process of membrane perforation protein, which is different from necroptosis and pyroptosis [50, 51] (Figure 1).

### 3.3. Indicators of Ferroptosis

Ferroptosis is accompanied by a series of molecular biological changes. Morphologically, transmission electron microscopy (TEM) observes that cells undergoing ferroptosis mainly manifest as volume reduction, mitochondrial density increase, ridge reduction or disappearance, and mitochondrial outer membrane disruption [52]. More recent evidence indicates ferroptosis is accompanied by decreased cell viability with cholecystokinin octapeptide (CCK-8). In terms of iron metabolism, the PGSK probe is considered to display the increase of cell iron concentration. Quantitative PCR (qPCR), Western blot, and immunoblotting are used to detect the decrease of ferritin expression and the increase of TFR1 and hepcidin expression. In terms of lipid metabolism, GPX4, SLC7A11, COX2, and ACSL4 are considered as specific biomarkers of ferroptosis. qPCR or Western blot show that GPX4 and SLC7A11 are significantly downregulated, while the expression of COX2 and ACSL4 increased. Mitochondrial function has been proposed as another ferroptosis biomarker. qPCR and Western blot suggest that DHODH is significantly upregulated in ferroptosis [53]. VDAC2 and VDAC3 are necessary and insufficient conditions for erastin-induced ferroptosis. Infection with shRNA targeting VDAC2 has been used as a positive control for ferroptosis research [3].

## 4. Differences and Connections between Ferroptosis and Other RCDs

### 4.1. Ferroptosis and Necrosis

Necrosis is a passive cell death under substantial physical and chemical or biological factors. It is morphologically characterized by increased cell swelling, organelle deformation or swelling, and eventually cell rupture. Necrotic cell division explains the release of inclusions and causes an inflammatory response, often accompanied by fibrosis of tissues and organs during healing. With detailed studies, multiple proteins participate in the signal regulation of necrosis. Ferroptosis is a newly described caspase-independent regulatory necrosis, featuring iron-dependent necrosis which resulted in lethal accumulation of lipid peroxides. In the glioblastoma (GBM) model, neutrophils transfers myeloperoxidase particles into tumor cells, inducing iron-dependent accumulation of lipid peroxides in tumor cells and promoting iron-dependent necrosis of glioblastoma [54]. Since ferroptosis is crucial regulatory necrosis, triggering ferroptosis to induce cancer cell necrosis plays an increasingly important role in the clinical treatment of malignant tumors.

### 4.2. Ferroptosis and Apoptosis

Apoptosis is a programmed cell death in that cells “end” their lives automatically under certain physiological or pathological conditions, controlled by an internal genetic mechanism, morphologically manifested as cell shrinkage and the formation of an apoptosome [55]. Under certain conditions, apoptosis and ferroptosis promote each other in positive feedback. p53, an apoptosis-related tumor suppressor gene, upregulates ferroptosis [56]. Ferroptosis further advances cells' sensitivity to apoptosis, while the ferroptosis inducer promotes the expression of the p53 upregulated modulator of apoptosis (PUMA) by protein-folding reaction [57, 58]. The above-mentioned results indicate the mutual promotion between ferroptosis and apoptosis, and it is of great significance in modulating ferroptosis by adjusting apoptosis in clinical antitumor therapy.

### 4.3. Ferroptosis and Autophagy

Autophagy is a process in which cytoplasmic proteins or organelles are phagocytized and coated into vesicles, forming autolysosomes and degrading contents, morphologically manifested as cytoplasmic vacuolization and autophagosome formation [59]. Most investigators concur that autophagy is a necessary step of ferroptosis and promotes the occurrence of ferroptosis. On the one hand, autophagy degrades ferritin, and ferritinophagy is a critical factor in ferroptosis [60]. Autophagy-dependent lysosome ROS formation stimulates ferroptosis and provides unstable iron through NCOA4-mediated iron autophagy [61]. On the other hand, intracellular Fe^2+^ and LPO reduction happened after targeted knockdown autophagy-related gene 5 (Atg5) and Atg7 genes [62, 63]. Thus, the induction of autophagy is necessary for ferroptosis, and ferroptosis is essentially a process of autophagy cell death [45, 64, 65]. Regulating ferroptosis by targeting autophagy-related pathways is potentially an essential method for clinically treating ferroptosis-related diseases.

### 4.4. Ferroptosis and Necroptosis

Necroptosis is a kind of cell death with similar morphological necrosis and signaling pathways of apoptosis [66], characterized by necrosis-like cell death [67]. Ferroptosis and necroptosis coincide under ferroptosis inducer erastin in human promyelocytic leukemia HL-60 cells [4]. Ferroptosis and necroptosis are mutually substituted and reciprocal. Ferroptosis resistance induces necroptosis, while necroptosis resistance promotes ferroptosis [68]. Inhibition of ferroptosis marker ACSL4 promotes mixed lineage kinase domain-like (MLKL) expression and downregulates MLKL-induced ferroptosis [68]. The murine embryonic fibroblast cell line (NIH3T3) with targeted MLKL gene knockdown shows specific resistance to necroptosis, and ferroptosis occurs immediately after treatment with erastin [69]. In addition, the change of intracellular NADPH content is correlated with the substitution between ferroptosis and necroptosis [70]. Therefore, targeted inhibition of MLKL to obtain necroptosis resistance enhances the clinical antitumor effect of the ferroptosis inducer to some extent.

### 4.5. Ferroptosis and Pyroptosis

Pyroptosis is a distinct RCD [71]. Pyroptosis is accompanied by the forming of caspase-1-dependent plasma membrane pores, leading to the release of proinflammatory cytokines and cell fragmentation, morphologically characterized by pyroptosis formation bodies [72]. The early ferroptosis stage shows a similar pattern to pyroptosis, which releases inflammatory injury-related factors to control the inflammatory response and coordinate antibacterial defense [73, 74]. Ferroptosis is a caspase-independent regulatory death. The mitochondrial permeability increased by Bcl-2-associated X protein/Bcl-2-associated K protein (Bax/Bak) is unnecessary [75]. Synergistic enhancement of antitumor activity and sensitivity to drug-resistant tumors happened when inducing pyroptosis and ferroptosis simultaneously [76]. Therefore, the synergistic antitumor effect of inducing pyroptosis and ferroptosis offers current knowledge to cancer treatment (Table 1).

## 5. Pulmonary Manifestations of Ferroptosis

### 5.1. Ferroptosis-Induced Inflammatory Response

The changes in the extracellular immune microenvironment play vital roles in the pathophysiology of lung diseases. COVID-19 is characterized by an invasive inflammatory response, which strongly leads to airway damage [77]. ALI undergoes alveolar epithelial dysfunction caused by acute inflammation and tissue injury [78]. COPD is associated with an abnormal inflammatory response induced by harmful particle deposition [79]. The research on the pathogenesis of idiopathic pulmonary fibrosis (IPF) mainly focuses on chronic inflammation [80]. The above provides evidence that lung inflammation participates in the development of lung diseases.

Lung inflammation is accompanied by lung cells' ferroptosis, which further promotes the inflammatory response. In the early stage of ferroptosis, cells release inflammatory injury-related factors to control the inflammatory response and coordinate host defense [73, 74]. Ferroptotic cells release damage-associated molecular patterns (DAMPs) and activate the advanced glycation end product specific receptor (AGER) to trigger the inflammatory response of peripheral macrophages and activate NF-*κ*B in innate immunity [46, 81]. Hemin, which is chemically similar to heme, causes ROS accumulation and lipid peroxidation in platelets through proteasome activity and inflammasome activation and induces cell ferroptosis [82]. Patients with COVID-19 showed high concentrations of proinflammatory CD4^+^ T cells and cytotoxic particle CD8^+^ T cells and dysregulation of iron homeostasis [83, 84]. The iron chelator lactoferrin (LF) inhibits iron overload to reduce inflammation and prevent SARS-CoV-2 from entering host cells [85]. The release of inflammatory factors in ALI enhances the accumulation of ROS and induces ferroptosis. The accumulated ROS functions as a positive regulator of inflammatory response and promotes the progression of ALI [86]. Early release of DAMPs from ferroptotic cells in COPD triggers an inflammatory response, and GPX4 knockdown favors the development of chronic inflammation induced by cigarette smoke [8]. Ferroptosis inhibitor ferrstatin-1 (Fer-1) inhibits the fibrogenic factor TGF-*β*1 during the progression of IPF to play the protective role against IPF [87]. Collectively, ferroptosis promotes lung inflammation through DAMPs and ROS, and targeted inhibition of inflammatory response mediated by lung cell ferroptosis is a potential strategy for lung disease therapy.

### 5.2. Ferroptosis in Pulmonary I/R Injury

Lung I/R injury (LIRI) is a common clinical-pathological phenomenon with high mortality, which can be caused by lung transplantation, atherosclerosis, and trauma [88–90]. Ischemia leads to an insufficient energy supply, intracellular calcium, and lactic acid accumulation and eventually cell death [91]. ROS and proinflammatory neutrophils after reperfusion increase lung tissue damage [91, 92]. Oxidative stress, inflammatory response, and toxic substances play crucial roles in the progression of lung disease during LIRI, in which NOX and labile iron are involved [93–95].

The LIRI process is accompanied by obvious ferroptosis of lung cells. Inefficient oxidative phosphorylation, ROS production in hypoxanthine metabolism, increase of labile iron caused by ischemia, and NOX expression can all trigger ferroptosis [89, 96]. In addition, LIRI contributes to the decrease of GSH and GPX4, the increase of ASCL4 expression, and the change of mitochondrial morphology, which favors the accumulation of lipid peroxidation [88]. Ferroptosis participates in the injury of the lung and other tissues and organs, which is related to the promotion of ferroptosis by I/R [97]. Ferroptosis is the primary form of cell death during IR [98], and ferroptosis of lung tissue cells aggravates LIRI. With detailed studies, ferroptosis exacerbates hepatic I/R injury, intestinal I/R injury, and ER stress-related myocardial I/R injury [6, 99, 100], and the induction of ferroptosis deteriorates I/R-related lung injury and pulmonary edema [97]. Most investigators concur that inhibiting ferroptosis can alleviate LIRI. Given the vital role of ferroptosis in I/R, diminishing ferroptosis and reversing LIRI are beneficial to COVID-19 treatment [101]. The inhibitor of the apoptosis-stimulating protein of p53 can inhibit ferroptosis and alleviate IR-induced ALI [102]. Upregulating Nrf2 attenuates ferroptosis and plays a protective role against intestinal IR-induced ALI [97]. In addition, pirfenidone ameliorates LIRI, ALI, and PF by modulation of inflammation and oxidative stress [103], indicating that the inhibition of ferroptosis may also be involved. In summary, LIRI is the pathological process of various lung diseases, and inhibiting ferroptosis alleviates LIRI-mediated lung diseases effectively.

### 5.3. Ferroptosis Triggers Immunological Changes

Normal lung function is closely related to immune homeostasis, and the congenital immune response is the first line of defense for early pulmonary infection [104]. Patients with COVID-19 have decreased lymphocytes, increased neutrophils [105, 106], and necrosis of the spleen, lymph nodes, and other lymphoid tissues, as well as lymphocyte infiltration in the alveolar septa [83, 106]. In ALI and PF, alveolar macrophages are activated [107], and neutrophils trigger the cascade of inflammation and fibrosis in the lungs [108]. A significant feature of COPD is inflammation, with increased macrophages, neutrophils, T and B lymphocytes, and dendritic cells (DC) [109]. Lymphocyte infiltration is associated with lung cancer [110].

Ferroptosis holds great therapeutic potential in many lung diseases related to ferroptosis-mediated immune changes in the lung and whole body. There are many immune cells, such as macrophages and DC in the lungs, which play a crucial role in maintaining the immune homeostasis of the lung [107]. The signals released by ferroptotic cells can recruit antigen-presenting cells (APCs), such as macrophages, DC, and neutrophils, to the site of ferroptosis and promote antigen presentation to initiate and regulate immune response [111, 112]. When ferroptosis occurs in lung tissue, the APCs mentioned above migrate to the damaged area [113] and start specific immunity by recognizing, phagocytizing, and directly killing the pathogen or promoting antigen presentation. At present, lung cancer has been mainly studied for ferroptosis-mediated immune changes. Ferroptosis favors the activation of macrophages [74], which is beneficial for pulmonary immune monitoring [114]. Nonetheless, GPX4 diminishes lipid peroxidation to maintain activation of T regulatory cells and attenuate antitumor immunity [115]. Current knowledge argues that interferon-*γ* (IFN*γ*) produced by immune cells downregulates SLC3A2 and SLC7A11 to trigger ferroptosis [116], and the induction of ferroptosis contributes to the antitumor efficacy of immunotherapy [117]. With detailed studies, an increase in tumor-associated macrophages (TAM) in the tumor microenvironment is associated with poor prognosis of patients with lung adenocarcinoma [117], and ferroptosis attenuates metabolic and inflammatory regulation of TAM to elicit antitumor activity [118]. Ferroptotic cancer cells release immunomodulatory molecules to induce systemic immune change, including DAMP, oxidized lipid mediators, and prostaglandin E2 (PGE2). DAMP triggers the toll-like receptor 4 (TLR4) signals of neutrophils and DC, activating the innate immune system [111]. Oxidized lipid mediators induce GPX4 depletion to promote T cell ferroptosis [119]; oxidized phosphatidylcholine inhibits DC maturation through Nrf2 activation and inhibits the differentiation of T helper cell 17 (Th17) [120]. PGE2 plays an essential role in both innate and regulatory immunity. PGE2 directly attenuates the function of cytotoxic T cells and conventional type 1 dendritic cells (cDC1) or diminishes the accumulation of cDC1 in tumor sites by inhibiting the natural killer (NK) cell-chemokine CC chemokine ligand 5 (CCL5)/chemokine lymphotactin (XCL1) pathway [25, 121].

Therefore, the immune response is crucial in the lungs and the whole body, and the pathophysiological process of lung diseases is accompanied by the ferroptosis of lung cells. Ferroptosis-mediated immune changes precisely regulate the progression of lung diseases. The regulation of ferroptosis in lung cells and ferroptosis-mediated immune changes holds broad prospects in treating lung diseases.

## 6. Ferroptosis and Lung Disease

### 6.1. Ferroptosis and COVID-19

**Figure 2:**
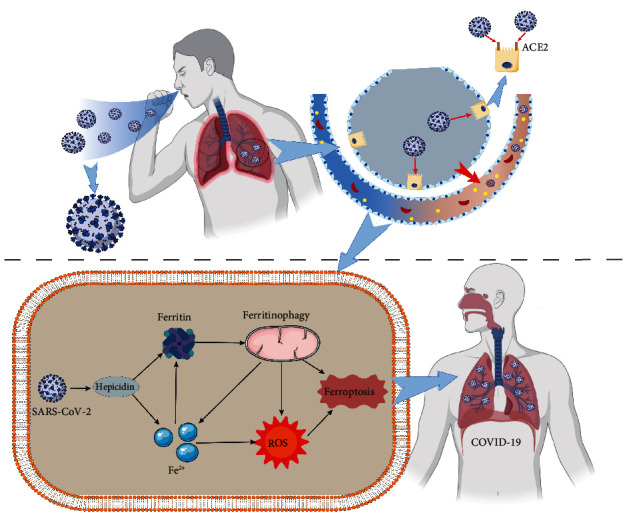


COVID-19 is a new type of fulminant epidemic disease caused by SARS-CoV-2 infection, clinically manifested with pneumonia, acute respiratory distress syndrome (ARDS), and sepsis, leading to multiple organ failure [122, 123]. More recent evidence indicates that SARS-CoV-2 induces iron overload by promoting the overexpression of hepcidin [124, 125]. COVID-19 infection leads to an inflammatory state, including cytokine storm, in which IL-6 stimulates the synthesis of ferritin and hepcidin. Both upregulation of hepcidin and downregulation of ferritin significantly impede iron output, leading to iron overload and ferroptosis in cells [126]. With detailed studies, SARS-CoV-2 simulates hepcidin to increase serum ferritin content significantly. Hyperferritinemia enriches high oxidative stress and LPO and ultimately increases mitochondrial autophagy leading to ferroptosis [127, 128]. The overexpression of hepcidin and iron overload play critical roles in COVID-19, and hepcidin has been proposed as a specific biomarker to measure the effectiveness of COVID-19 therapy. Studies have indicated that a large amount of phospholipid oxide accumulates in myocardial cells of COVID-19 patients. Ferroptosis aggravates myocardial I/R injury, which contributes to myocardial injury and multiple organ failure of COVID-19 [129–131]. Overall, ferroptosis may take a vital part in promoting COVID-19, and inhibition of ferroptosis sheds new light on reliable treatments of COVID-19 (Figure 2).

### 6.2. Ferroptosis and ALI

ALI is an acute and fatal illness after severe infection, trauma, and harmful gas inhalation, associated with pathological features, namely, pulmonary edema and atelectasis caused by diffuse alveolar-capillary membrane injury. The clinical manifestations are respiratory distress and refractory hypoxemia [132]. Of note, uncontrolled lung inflammation and oxidative/antioxidant imbalance are the two main mechanisms in the complex pathogenesis of ALI [133]. Ferroptosis is reportedly crucial in various ALI models, including lipopolysaccharide- (LPS-) induced ALI [134], intestinal I/R injury-induced ALI [102], seawater drowning-induced ALI [135], and oleic acid-induced ALI [136], as well as pseudomonas aeruginosa- (PA-) induced ALI [137]. The development of ALI stimulates ferroptosis, which further aggravates ALI [9].

In multiple ALI models, the release of inflammatory cytokines contributes to oxidative damage and uncontrolled inflammation and increases intracellular lipid ROS, myeloperoxidase (MPO), and malondialdehyde (MDA) levels which confer resistance to GSH content GPX4 activity, eventually leading to severe mitochondrial damage, presented as obvious ferroptosis [138, 139]. Inflammatory out-of-control and cytokine storm in ALI facilitated ROS accumulation in lung tissue, which further boosted ferroptosis [134, 140]. ROS-induced oxidative damage and inflammatory activity in ferroptosis further promote the release of inflammatory factors and aggravate LPS-induced ALI [141]. Ferroptosis and LPS-induced ALI show positive feedback to each other. Thus, inhibition of ferroptosis is expected to be useful in ALI therapy.

Nrf2 functions as a positive regulator of ALI by inhibiting ferroptosis. With detailed studies, exogenous Nrf2 agonist dimethyl fumarate (DMF) promotes nuclear translocation of Nrf2 through the p62-Keap1-Nrf2 axis [135]. Nrf2 enhances the inhibition of ferroptosis-related genes HIF-1*α*, HO-1, and SLC7A11 by reducing GSH loss; inhibiting MDA, ROS, and lipid ROS accumulation; and enhancing mitochondrial function to diminish ferroptosis and alleviate ALI [86, 142]. HIF-*α* plays a vital role in upregulating antiferroptotic defense, which resists iron overload and increases GPX4 expression [143]. HO-1, a ROS detoxifying enzyme, has a protective effect against oxidative stress by upregulating the Nrf2/ARE pathway [144]. The SLC7A11 gene encodes cystine/glutamate xCT transporter, which reduces oxidative stress in epithelial cells by increasing the intracellular cystine level and inhibits the Nrf2/HO-1 signaling pathway by negative feedback to maintain cellular antioxidant balance [142]. Nrf2/HIF-1*α* and Nrf2/HO-1/SLC7A11 signaling pathways have recently attracted widespread attention in inhibiting ferroptosis and alleviating ALI [102, 145].

**Figure 3:**
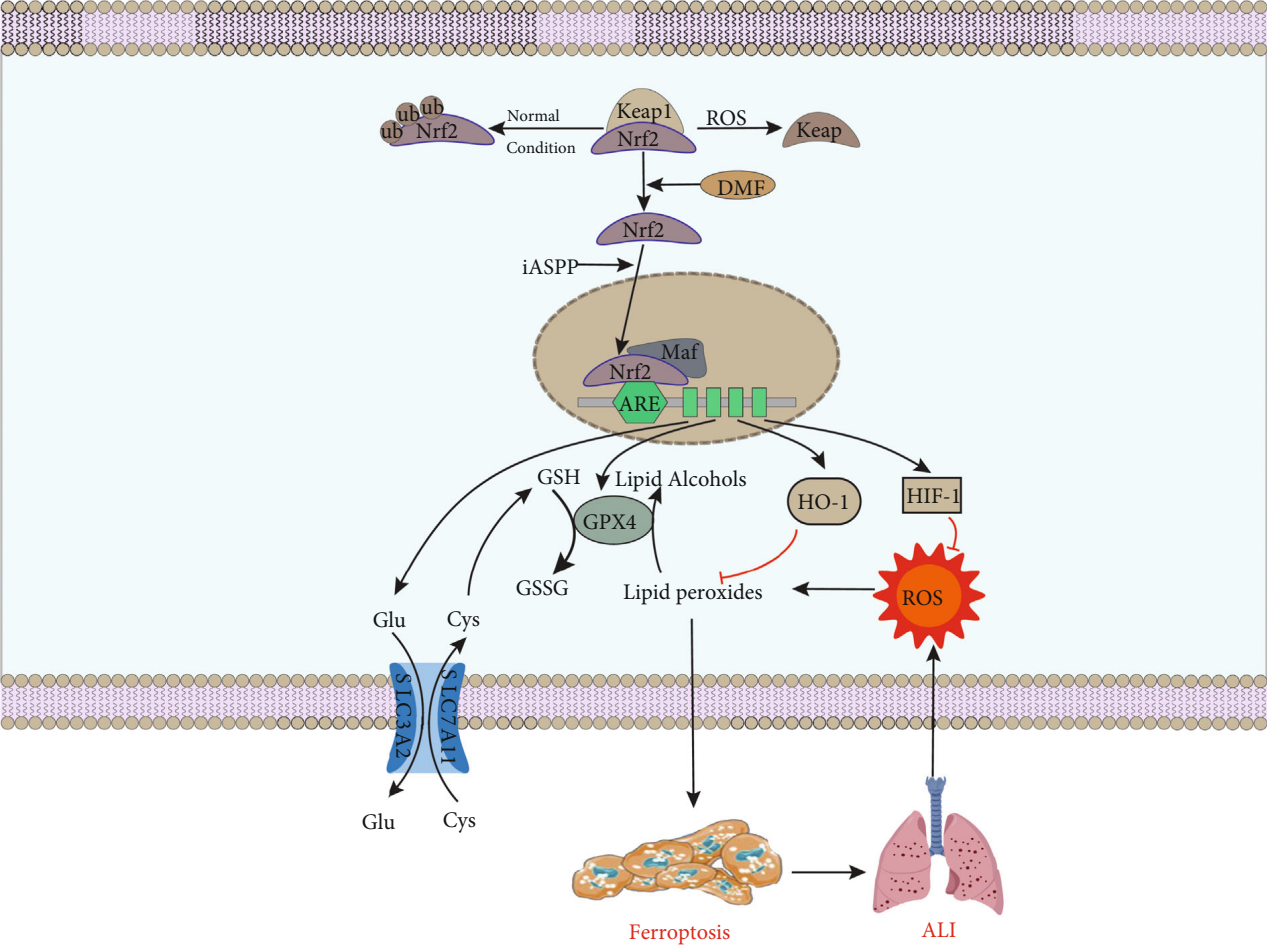


The inhibitor of apoptosis stimulating p53 protein (iASPP) is a p53 inhibitor that inhibits ferroptosis by promoting Nrf2 aggregation and nuclear translocation and further alleviates ALI [146, 147]. Fer-1 attenuates ALI by inhibiting LPO and reducing total iron levels in cells [148]. Thus, inhibition of ferroptosis in lung tissue is a promising therapy of ALI (Figure 3).

### 6.3. Ferroptosis and PF

PF is a chronic progressive interstitial lung disease accompanied by the proliferation of myofibroblasts [149]. Recent studies indicate that ferroptosis in lung tissue promotes the development of PF. In the IPF model, erastin decreases the activity of GPX4 in human fetal lung fibroblasts and contributes to the accumulation of lipid peroxides, *α*-SMA, COL1, and fibroblast differentiation [150]. Ferroptosis inhibitor Fer-1 enhances GPX4 expression and reduces LPO. Moreover, Fer-1 eliminates the effect of TGF-*β*1 and erastin on cell ferroptosis [87], inhibits the differentiation of fibroblasts into myofibroblasts, and delays the process of PF.

**Figure 4:**
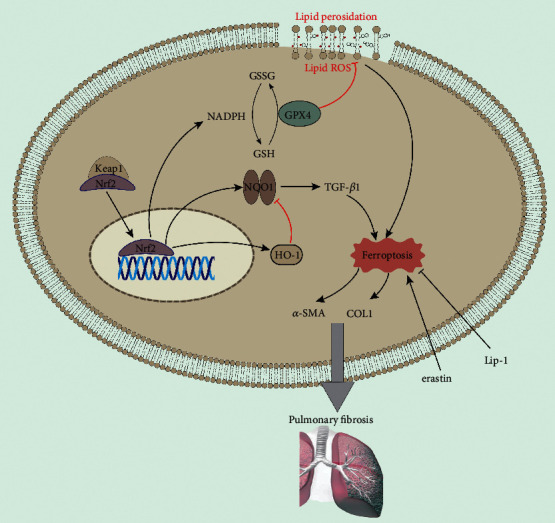


Ferroptosis promotes the occurrence and development of radiation-induced lung fibrosis (RILF) [151]. In the irradiation group in the RILF mouse model, the volume of lung tissue decreases, the mitochondrial density increases, the crista reduces, and the outer membrane is interrupted, showing typical ferroptosis characteristics. Ferroptosis inhibitor liproxstatin-1 (Lip-1) has a significant therapy effect on RILF mice. Lip-1 can increase GPX4 level, inhibit collagen deposition, and reduce inflammatory cytokines and ROS in RILF mice [10]. In addition, Lip-1 attenuates RILF by activating Nrf2, promoting HO-1 and NQO1 expression, downregulating TGF-*β*1, and inhibiting ferroptosis in lung tissue [10]. Consequently, these findings provide evidence for the clinical treatment of PF by inhibiting ferroptosis (Figure 4).

### 6.4. Ferroptosis and COPD

COPD is chronic bronchitis or emphysema, characterized by airflow obstruction, further developing into a common chronic disease of pulmonary heart disease and respiratory failure. COPD is associated with abnormal inflammation caused by harmful gases and particle deposition, with high morbidity and mortality [79]. Although an initial study indicates that ferroptosis occurs in the lung tissue of COPD mice, the current knowledge on the integrated molecular mechanism underlying ferroptosis-involved COPD is controversial.

Some investigators concur that chronic inflammation caused by long-term smoking is one of the pathogenesis of COPD [152]. At the core of the process, free radicals in cigarette smoke induce cell iron overload, promote ferroptosis, and accelerate the development of COPD [153]. Studies have shown that cigarette smoke induces ferroptosis in bronchial epithelial cells by promoting endoplasmic reticulum stress and mitochondrial homeostasis disorder [154]. NCOA4 is a selective receptor for autophagy recognition of ferritin. Cigarette smoke increases the expression of NCOA4, thereby increasing the selective autophagy of ferritin to trigger iron overload and inducing ferroptosis [61]. Moreover, cigarette smoke increases 4-hydroxy-2-nominal (4-HNE) production and downregulates GPX4 expression to accelerate intracellular LPO [8]. DAMPs are released early in ferroptosis to promote chronic inflammation during COPD progression [155].

**Figure 5:**
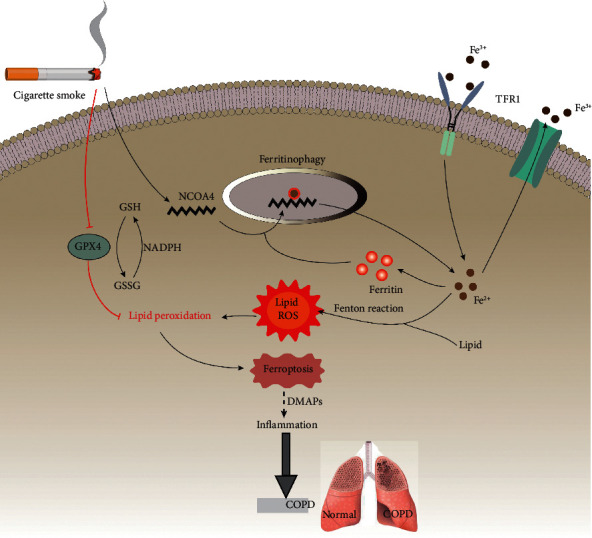


Inhibition of ferroptosis has a good prospect in the clinical treatment for COPD. Ferroptosis inhibitors, deferoxamine and Fer-1, alleviate cigarette smoke-induced ferroptosis in bronchial epithelial cells [153]. Targeting lipid metabolism-related DAMP signaling might be a promising strategy for treating COPD related to ferroptotic damage (Figure 5).

### 6.5. Ferroptosis and Lung Cancer

Lung cancer is the leading cause of death worldwide in males and females [156], usually divided into small cell lung cancer (SCLC) and non-small cell lung cancer cells (NSCLC). Currently, the clinical treatments of lung cancer are mainly chemotherapy drugs and targeted drugs, which achieve the therapeutic purpose by inducing apoptosis [157], but limitations in application still exist. Cancer cells require higher iron metabolism levels and are more susceptible to ferroptosis [158]. In general, ferroptosis resistance is widespread in cancer cells, and inducing ferroptosis in lung cells is expected to become a new target for clinical treatment of lung cancer [159, 160].

#### 6.5.1. The Antiferroptotic Defense of Lung Cancer Cells

Inhibition of p53 activity is prevalent in tumor cells [161], and acetylation is crucial to p53-mediated ferroptosis and tumor inhibition [43]. SLC7A11 and SLC3A2 are highly expressed in tumor cell lines and increase cellular antioxidant capacity. High expression of serine-threonine tyrosine kinase 1 (STYK1) in NSCLC cell line SW900 promotes the expression of GPX4 and the proliferation of lung cancer cells [162]. In addition, high expression of FSP1 in lung cancer cells promotes lipophilic antioxidant production and inhibits ferroptosis [33, 34].

Iron metabolism is closely related to the tumor microenvironment (TME), which accelerates tumor progression by promoting DNA replication [163, 164]. More recent evidence indicates that high ferritin expression in lung cancer tissues of NSCLC patients attenuates the Fenton reaction to reduce ferroptosis. Iron-sulfur cluster synthase 1 (NFS-1) is generally highly expressed in lung cancer tissue and cell lines. Moreover, NFS-1 collects more sulfur elements from Cys to produce iron-sulfur clusters, which diminishes the release of iron from cells and significantly alleviates the high oxygen-induced cell ferroptosis [165]. Hepcidin and ferroportin-1 (FPN1) constitute important regulators of iron homeostasis *in vivo*. Indeed, increased plasma hepcidin promotes tumor progression by inhibiting FPN1-induced NF-*κ*B and Wnt signaling pathways and iron-dependent ROS production.

**Figure 6:**
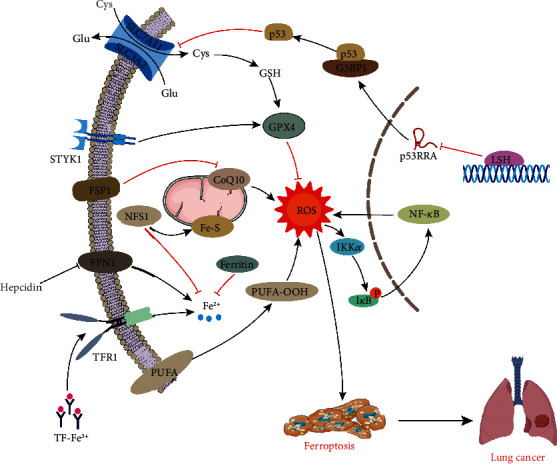


Lung cancer cells fight against ferroptosis by regulating lipid metabolism through lymphoid-specific helicase (LSH). As a DNA methylation modifier, LSH inhibits ferroptosis by activating lipid metabolism-related genes, including glucose transporter 1 (GLUT1), ferroptosis-related genes sterol-CoA desaturase 1 (SCD1), and fatty acid desaturase 2 (FADS2). Thus, LSH is a novel antiferroptotic molecule, which inhibits ferroptosis by reducing intracellular iron and ROS levels [166, 167] (Figure 6).

#### 6.5.2. Ferroptosis and Lung Cancer Treatment

There is a broad prospect of treating lung cancer by using a ferroptosis inducer [160]. Cisplatin is an inducer of ferroptosis and apoptosis in NSCLC A549 cells, promoting the depletion of reduced GSH and inactivation of GPX4. Ginkgo flavonoids enhance cisplatin's efficacy in treating NSCLC by downregulating the Nrf2/HO-1 pathway [142]. GPX4 inhibitor enhances cisplatin's antitumor activity, and cisplatin combined with erastin has a significant synergistic effect on antitumor activity [168, 169]. Erastin inhibits cell proliferation, promotes G2/M phase arrest, triggers iron transport, and inhibits cell migration in lung cancer cells. Natural product erastin has the potential of treating lung cancer by upregulating p53 expression and promoting the Ca^2+^/CaM pathway to induce ferroptosis [169–174]. In addition, erastin combined with acetaminophen (APAP) inhibits NSCLC cell viability and promotes ferroptosis and apoptosis by suppressing Nrf2 nuclear translocation [175]. The lysosomal instability drug siramesine and dual tyrosine kinase inhibitor lapatinib induce ferroptosis in A549 cells by decreasing the HO-1 level [176]. Dihydroartemisinin (DHA) inhibits lung cancer cell proliferation and colony formation, enhances cell death, and induces ferroptosis by inactivating the polypeptide 2 (PRIM2)/SLC7A11 axis, indicating that the inhibitory expression of PRIM2 may be a potential therapeutic target for lung cancer [177]. Ferroptosis induced by STYK1 inhibits the development of cancer cells. Zinc poisoning promotes ferroptosis in cancer cells and upregulates antiferroptotic defense [178], which indicates that targeting ferroptosis has been mounting interest in the treatment for NSCLC [162].

Long noncoding RNA (lncRNA) LINC00336 reduces iron concentration, lipid ROS, and mitochondrial superoxide and increases mitochondrial membrane potential, consistent with its role in ferroptosis [179]. Metallothionein 1D pseudogene (MT1DP) is a lncRNA that intensifies oxidative stress by inhibiting the antioxidant effect of Nrf2 [180]. P53RRA is a standard tumor suppressor that inhibits lung cancer by activating the p53 pathway and promoting ferroptosis [181]. LSH is directly recruited into the promoter region of p53RRA to silence p53RRA, and DNA methylation is involved in the inhibition of p53RRA. Ferroptosis is epigenetically regulated by LSH and promotes lipid metabolism genes, including SCD1 and FADS2 expression [167]. The prospect of ferroptosis induction in cancer treatment is growing, but its therapeutic effect is low due to the reduction of Fenton reaction in the TME. Mixed semiconductor nanozymes (HSN) produce heat under light to enhance cytotoxicity and Fenton reaction and increase hydroxyl radical production to promote ferroptosis in tumor cells [182].

After tumor immunotherapy, IFN*γ* released by activated CD8 ^+^ T cells downregulates the expression of SLC7A11 and SLC3A2, thereby inhibiting the uptake of cystine in tumor cells and enhancing LPO and ferroptosis. Inhibition of synergistic effect of system Xc^−^ and GPX4 by ferroptosis inducers and radiation promotes ferroptosis by enhancing LPO in the cytoplasm. Ferroptosis inducer may be an effective radiosensitizer to expand the efficacy and indications of radiotherapy [183]. Drug resistance has become a significant problem in the chemotherapy treatment of lung cancer. The combined use of ferroptosis inducers and chemotherapy drugs may become a new strategy for lung cancer treatment. After combined treatment, tumor cells showed typical morphological changes of ferroptosis [168]. The magnetic field (MF) induces oxidative stress and activates the DNA damage repair pathway through ROS-induced DNA damage, eventually leading to apoptosis and ferroptosis and inhibiting tumor growth [184]. MF is being used for antitumor treatment, but its potential biological mechanism is still unclear.

Clinical treatment of patients with tumor metastasis is a global problem. Targeted ferroptosis may be a promising method to prevent lung cancer metastasis [185]. Compared with local injections, nanoparticles have more tremendous advantages in treating metastasis-associated cancer with relatively low risk [186]. Based on the coordination between ferric ion (Fe^3+^) and tannic acid (TA), a p53 plasmid encapsulated metal-organic network (MON-p53) is constructed. MON-p53 plasmid inhibits the growth of cancer cells by promoting ferroptosis. The MON-p53 complex has broad prospects in targeting ferroptosis of tumor cells [56]. Lung cancer cells are prone to metastasis and drug resistance. The antagonistic Merlin-yes-associated protein (YAP) pathway promotes ferroptosis by upregulating ferroptosis regulators such as ACSL4 and transferrin receptor (TFRC) [187]. Together, these findings provide evidence that metastatic cancer cells are susceptible to ferroptosis, which indicates excellent treatment prospects in lung cancer by upregulating ferroptosis (Table 2).

### 6.6. Ferroptosis and Other Lung Diseases

#### 6.6.1. Ferroptosis and Asthma

Ferroptosis has been implicated in the pathogenesis of asthma recently. The phosphatidylethanolamine-binding protein 1/15 lipoxygenase (PEBP1/15-LO) complex plays a vital role in regulating ferroptosis. Compared with regular patients, there were increased colocalization levels of PEPBP1 and 15-LO in human airway epithelial cells (HAECs) of asthmatic patients, which suggests that ferroptosis may occur in asthma. Blocking PEBP1 in HAECs decreases the sensitivity to ferroptosis [188]. Moreover, the 15LO1-PEBP1-generated ferroptotic phospholipid, 15-hydroperoxy-arachidonoyl-phosphatidylethanolamine (15-HpETE-PE), promoted light chain3-I (LC3-1) to stimulate autophagy. In type 2 Hi asthma, there were high levels of both 15LO1-PEBP1 and LC3-II in HAECs, which are associated with low bronchoalveolar lavage fluid mitochondrial DNA and more severe disease [189]. Therefore, inhibiting ferroptosis in HAECs may be an effective asthma treatment.

#### 6.6.2. Ferroptosis and Allergic Airway Inflammation

Allergic airway inflammation (AAI) is a chronic inflammation characterized by increased airway responsiveness to various stimuli, which causes recurrent asthma, chest tightness, and cough [190]. Eosinophils play a vital role in the development of AAI. After ferroptosis-inducing agent (FIN) treatment, eosinophils shrink and mitochondria are damaged, showing typical ferroptosis morphology [3, 191]. Further study suggests that erastin and RSL3 inhibit GSH production in eosinophils and decrease GPX4 activity, while artesunate (ART) induces ferroptosis in eosinophils by promoting Fenton reaction [25, 192]. In addition, FINs combined with dexamethasone increase ROS production in the cytoplasm, induce eosinophilic ferroptosis, and alleviate AAI [193]. Thus, it is feasible to treat AAI by inducing eosinophilic ferroptosis.

#### 6.6.3. Ferroptosis and Infectious Lung Diseases

Ferroptosis plays an adverse role in infectious lung diseases. Pseudomonas aeruginosa (PA), a prokaryotic bacterium, can express lipoxygenase (pLoxA) to oxidize host acid-phosphatidylethanolamines (AA-PE) to 15-hydroperoxy-AA-PE (15-HOO-AA-PE), triggering ferroptosis in human bronchial epithelial cells [194]. Further study suggested that PA can activate the lysosomal chaperone-mediated autophagy (CMA) to decrease the host GPX4 defense. Meanwhile, the host can stymie lipid peroxidation and protect GPX4/GSH-deficient cells by stimulating the inducible nitric oxide synthase (iNOS)/NO-driven antiferroptotic mechanism [195]. Therefore, promoting the iNOS/NO-driven antiferroptotic mechanism may provide a new strategy for the host against PA-induced ferroptosis. In addition, mycobacterium tuberculosis (Mtb) can cause macrophage necrosis, which is associated with reduced GSH and GPX4, with increased lipid peroxidation, mitochondrial superoxide, and free ion. Moreover, Fer-1 treatment can inhibit necrotic cell death in Mtb-infected macrophage and pulmonary necrosis in acutely infected mice and reduce bacterial load in infected animals [196]. These findings suggest that ferroptosis plays an essential role in Mtb infection and can be a novel target for the treatment of tuberculosis.

#### 6.6.4. Ferroptosis and Radiation-Induced Lung Injury

Radiation-induced lung injury (RILI) is a severe and life-threatening complication of thoracic radiotherapy, including acute radiation pneumonitis and RILF [197]. ROS accumulation induced by irradiation in lung tissue is a critical factor in the pathogenesis of RILI, which is also the leading cause of ferroptosis. In acute RILI mice, the level of ROS is upregulated and the level of GPX4 is downregulated in the lungs, which is reversed by treatment with Lip-1. Moreover, treated with Lip-1, RILI mice exhibit lower serum levels of inflammatory cytokines, mitigatory structure damage, and hemorrhage and declined mitochondria shrinkage [198]. Together, ferroptosis plays an essential role in acute RILI, and inhibition of ferroptosis may be an effective therapy for acute RILI.

## 7. Conclusion and Foresight

The exploration on the fine mechanism of regulating ferroptosis is developing rapidly, and the research on ferroptosis is being ushered in an outbreak. LPO accumulation and iron dependency contribute to ferroptosis, which is distinct from other types of RCDs. Recent studies indicate that cell ferroptosis sensitivity is regulated by the extrinsic or transporter-dependent pathway and the intrinsic or enzyme-regulated pathway (SLC7A11/GPX4, NADPH/FSP1/CoQ10, and GCH1/BH4), and the sensitivity of lung cells to ferroptosis is closely related to the progression of lung diseases. With detailed studies, the pathological process of lung diseases is accompanied by ferroptosis in lung cells, which promotes lung inflammation and lung I/R injury, and mediates lung and systemic immune changes. The targeted inhibition of ferroptosis alleviates lung inflammation and lung I/R injury, which is beneficial to clinical treatment of lung diseases such as COVID-19, ALI, PF, and COPD. In addition, inducing ferroptosis in lung cancer cells and enhancing antitumor immunity have broad prospects in the diagnosis and treatment of lung cancer. Many precise targets for inducing and inhibiting ferroptosis have been identified, but the targeted regulation of ferroptosis for lung diseases still needs to be further explored.

The research progress on the ferroptosis mechanism is rapid, and opportunities and challenges coexist. Ferroptosis is one of the crucial RCDs that regulate the body's environment's stability and plays a vital role in human health. In the early stage, combined and regular application of ferroptosis inducers and control of appropriate doses are conducive to improving the specificity of ferroptosis inducers and enhancing the effect of regulating ferroptosis in treating pulmonary diseases. The heterogeneity and plasticity of cancer cells may lead to the variation of ferroptosis sensitivity. It is necessary to hold the sensitivity of cancer cells to ferroptosis to avoid tumor escape. The regulation of endogenous Fe^2+^, GSH, GPX4, NADPH, PUFAs, and lipid antioxidants has been proposed as the key of ferroptosis, further clarifying that the molecular mechanism of the different molecular regulations of ferroptosis remains a challenge.

With further understanding of the mechanism of ferroptosis, it is possible to improve lung diseases' therapeutic effect by simultaneously regulating multiple cell death pathways. The occurrence of ferroptosis may be influenced by some immune processes, whose emerging regulatory network may be crucial in lung disease therapy. It is still in the infancy of the research on targeted therapy and drug design of lung diseases based on ferroptosis regulation. We may focus on targeting drugs and biological carriers' development in future work to make it a historic breakthrough in the clinical treatment of lung diseases through interdisciplinary cooperation.

## Figures and Tables

**Table 1:** Comparison of characteristics of ferroptosis and necrosis, apoptosis, autophagy, necroptosis, and pyroptosis.

Cell death manner	Morphologic features	Biochemical characteristics	Key controlling genes	References
Ferroptosis	Membrane rupture or blistering, mitochondrial atrophy, crista reduction, lack of chromatin condensation	Lipid peroxidation in cells induced by ferrous or esterase	GPX4, p53, Ras, Nox, SLC7A11, TFR1	[3, 4, 52]
Necrosis	Lipid peroxidation in cells induced by ferrous or esterase	Inflammatory response	—	
Apoptosis	Cell membrane foaming, cell contraction, the formation of apoptotic bodies	DNA degradation	Bcl-2 family, caspase family, C-myc, p53	[55, 56]
Autophagy	Cytoplasmic vacuolization to form autophagosomes	Increase activity of lysosomes	ATG5, ATG7, Beclin1	[59, 199]
Necroptosis	Cell volume enlargement, organelle swelling, cell membrane perforation, with necrotic cell characteristics	Cell collapse, release contents, triggering an immune response, and clearing the dead cells through macropinocytosis bodies	RIPK1, RIPK3, TNFR1, caspase-8	[66, 67]
Pyroptosis	Cell swelling, with a large number of bubble-like protrusions; a large number of vesicles, pyroptosis bodies, formed before the rupture of the plasma membrane	Formation of inflammatory bodies, activation of caspase and gasdermin, the release of a large number of proinflammatory factors	Caspase family, NLRP3, ASC	[71–73]

**Table 2:** The role and mechanism of ferroptosis in pulmonary diseases.

Disease	Research object	Biochemical features	Regulation mechanism	Inhibitors/inducers	References
COVID-19	Serum	Thrombosis, accumulation of oxidized phospholipids	Hepcidin	—	[124, 125, 130]
ALI	Human bronchial epithelial cells BEAS-2B and mouse lung cells	Lung inflammation out of control, oxidation/antioxidant imbalance	Nrf2/HIF-1, Nrf2/HO-1/SLC7A11, p62/Keap1/Nrf2 axis, PTGS2	Inhibitor Fer-1	[102, 133, 135, 137, 145]
PF	Human fetal lung fibroblasts HFL1, RILF mouse lung tissue cells	Increased ROS, lipid peroxidation, and fibroblast differentiation	*α*-SMA, COLI, Nrf2/HO-1/NQO1	Inhibitor Fer-1, Lip-1	[87, 151]
COPD	Human bronchial epithelial cells and mouse mode	Lipid peroxidation increased production of 4-HNE and DAMPs	NCOA4, GPX4	Inhibitor, deferoxamine, and Fer-1	[154, 155]
Lung cancer	NSCLC cell SW900, human plasma	Upregulation of GPX4/GSH pathway, reduction of iron, inhibition of lipid synthesis, and upregulation of FSP1	NF2-YAP, ACSL4, PRIM2/SLC7A11 axis, Nrf2/HO-1 axis, Ca^2+^/CaM, P53RRA	Inducer cisplatin, erastin, DHA, STYK1	[142, 169–174, 177, 178, 187]

## Abbreviations

15-HOO-AA-PE: 15-Hydroperoxy-AA-PE
15-HpETE-PE: 15-Hydroperoxy-arachidonoyl-phosphatidylethanolamine
AAI: Allergic airway inflammation
AA-PE: Acid-phosphatidylethanolamines
ACSL4: Acyl-CoA synthetase long-chain family member 4
AGER: Advanced glycation end product specific receptor
AHCYL1: Adenosylhomocysteinase like 1
ALI: Acute lung injury
APAP: Acetaminophen
APCs: Antigen-presenting cells
ARDS: Acute respiratory distress syndrome
ART: Artesunate
Atg5: Autophagy-related gene 5
Bax/Bak: Bcl-2-associated X protein/Bcl-2-associated K protein
BH4: Tetrahydrobiopterin
CCK-8: Cholecystokinin octapeptide
CCL5: Chemokine CC chemokine ligand 5
cDC1: Conventional type 1 dendritic cells
CMA: Chaperone-mediated autophagy
COPD: Chronic obstructive pulmonary disease
CoQ10: Coenzyme Q10
COVID-19: 2019 coronavirus disease
CYB5R1: Cytochrome b5 reductase
Cys: Cysteine
DAMPs: Damage-associated molecular patterns
DC: Dendritic cells
DCYTB: Duodenal cytochrome B
DHA: Dihydroartemisinin
DMF: Dimethyl fumarate
DMT1: Divalent metal transporter
ESCRT-III: Endosomal sorting complex required for transport-III
ETC: Electron transport chain
FADS2: Fatty acid desaturase 2
Fer-1: Ferrstatin-1
FINs: Ferroptosis-inducing agents
FPN1: Ferroportin-1
FSP1: Ferroptosis suppressor protein 1
FTH1: Ferritin heavy chain 1
GBM: Glioblastoma
GCH1: Guanosine triphosphate cyclohydrolase 1
GLUT1: Glucose transporter 1
GPX4: Glutathione peroxidase 4
GSH: Glutathione
HAECs: Human airway epithelial cells
HMGB1: High mobility group protein B1
HO-1: Heme oxygenase 1
HSPB1: Heat shock protein beta-1
I/R: Ischemia-reperfusion
iASPP: Inhibitor of apoptosis stimulating p53 protein
IFN*γ*: Interferon-*γ*
iNOS: Inducible nitric oxide synthase
IPF: Idiopathic pulmonary fibrosis
LC3-1: Promoted light chain3-I
LF: Chelator lactoferrin
Lip-1: Liproxstatin-1
LIRI: Lung I/R injury
lncRNA: Long noncoding RNA
L-OH: Lipid alcohols
LOXs: Lipoxygenases
LPCAT3: Lysophosphatidylcholine acyltransferase 3
LPO: Lipid peroxidation
LPS: Lipopolysaccharide
LSH: Lymphoid-specific helicase
MAPK: Mitogen-activated protein kinase
MDA: Malondialdehyde
MF: Magnetic field
MLKL: Mixed lineage kinase domain-like
MON: Metal-organic network
MPO: Myeloperoxidase
MT1DP: Metallothionein 1D pseudogene
Mtb: Mycobacterium tuberculosis
MtFt: Mitochondrial ferritin
NCOA4: Nuclear receptor coactivator 4
NFS-1: Iron-sulfur cluster synthase 1
NK: Natural killer
NOX: NADPH oxidase
NQO1: NAD(P)H:quinone oxidoreductase 1
Nrf2: Nuclear factor erythroid-2-related factor
NSCLC: Non-small cell lung cancer cells
p53: Protein 53
PA: Pseudomonas aeruginosa
PEBP1/15-LO: Phosphatidylethanolamine-binding protein 1/15 lipoxygenase
PF: Pulmonary fibrosis
PGE2: Prostaglandin E2
pLoxA: Lipoxygenase
POR: P450 reductase
PUFAs: Polyunsaturated fatty acids
PUMA: p53 upregulated modulator of apoptosis
qPCR: Quantitative PCR
RCDs: Regulated cell deaths
RILF: Radiation-induced lung fibrosis
RILI: Radiation-induced lung injury
ROS: Reactive oxygen species
RSL3: RAS selective lethal 3
RTA: Radical-trapping antioxidant
SAHH: S-Adenosyl-L-homocysteine hydrolase
SARS-CoV-2: Severe acute respiratory syndrome coronavirus-2
SAT1: Spermidine/spermine N1-acetyltransferase
SCD1: Sterol-CoA desaturase 1
SCLC: Small cell lung cancer
STEAP3: Six-transmembrane epithelial antigen of prostate 3
STYK1: Serine-threonine tyrosine kinase 1
System Xc^−^: Cystine/glutamate antiporter
T: Helper cell 17
TA: Tannic acid
TAM: Tumor-associated macrophages
TCA: Tricarboxylic acid
TEM: Transmission electron microscopy
TF: Transferrin
TFR1: Transferrin receptor protein 1
TFRC: Transferrin receptor
TGF-*β*1: Transforming growth factor beta 1
TLR4: Toll-like receptor 4
Th17: T helper cell 17
TME: Tumor microenvironment
VDAC2/3: Voltage-dependent anion channel
XCL1: Chemokine lymphotactin
YAP: Yes-associated protein.
